# Iodine

**Published:** 1828-03

**Authors:** 


### IODINE.

*Cases of Enlargement of the Liver and Spleen, treated with Iodine.*

Case I.—Henry Baynes, æt. five years and five months, of a slight figure and scrofulous diathesis; admitted a patient at the Royal Universal Infirmary for Children, August 25th, under the care of Dr. Milligan. Complains of a tumor occupying the right hypochondrium, extending anteriorly in a line with the linea alba, and inferiorly to the superior part of the hypogastric region. It is firm, colourless, it pushes out the false ribs considerably, and is attended with shooting pains occasionally. Pulse regular; skin cool; bowels open twice a day; passes his urine in bed. His mother first perceived the tumor six months since, which has gradually increased to its present size. He has been under the care of several medical men, and has used mercury for it.

℞. Tinct. Iodinæ gtt. xlviij.; Aquæ fontanæ ℥vss.; Syrupi simp. ℥ss. Capiat cochl. amplum nocte maneque.

August 28th.—Shooting pains removed; tumor appears a little diminished; pulse regular; appetite better; bowels open twice a day; stools of a dark brown colour.

℞. Hydr. Submur. Sub. gr. ij.; Rhei Pulv. gr. iv. M. fiat pulvis statim sumendus.—Repet^r Mistura Tinct. Iodinæ.

September 18th.—Tumor continues to diminish; general health improves. Has ceased to pass his urine in bed.

Cont^r Mistura Tinct. Iodinæ.

[Had an attack of catarrh, during which the Iodine was intermitted for a short time.]

November 3d.—Tumor much softer, and continues to diminish in size; appetite good; sleeps well; pulse regular; skin cool; tongue clean and moist; bowels open.

℞. Cascarillæ Pulv. gr. v.; Ferri Tartarizati gr. iv. M. habeat xij. tales. Capiat unam ter die.

16th.—No tumor felt on examination of the soft parts of the abdomen; the false ribs still project, but general health is entirely restored. Discharged cured.

Case II.—Elizabeth Howard, æt. twenty months.

June 19th.—Was admitted a patient at the Universal Infirmary for Children, last week, for tertian ague, at the same time with her brother, who laboured under the same disease. Mr. Woodham, the house-surgeon, saw them, and prescribed some bark, which has removed the intermittent. A large tumor is perceptible in the left hypochondrium, extending to the linea alba anteriorly, and lower part of the iliac region inferiorly. Her mother observed this swelling commence about eight months since, after an attack of ague, and it has gradually acquired its present size; pulse small and weak; appetite bad; no thirst; five stools yesterday, of a dark green colour.

August 18th.—Mercury used externally and internally, with little intermission, up to this period, without any improvement in the tumor, Dr. Milligan determined on giving the tincture of iodine.

℞. Tinct. Iodinæ gtt. xiij.; Aquæ font. ℥jss.; Syrupi simpl. ℥ss. Dentur cochl. duo min. nocte maneque.

25th.—Tumor considerably diminished in size and become softer.

Repet^r mistura.

Sept. 1st.—Tumor reduced to a very small size and feels soft.

11th.—The enlargement of the spleen has almost disappeared, but another tumor is felt in the pubic region, the margins of which are altogether unconnected with the spleen: this seems to depend on some mesenteric glands being very much enlarged.

Repet^r mistura; augenda dosem ad gtt. iv. bis die.

18th.—Tumor in pubic region diminishes; general health very good.

Repet^r mistura; augendo dosem ad gtt. v. bis die.

22d.—Both tumors removed; general health continues good.

Repet^r mistura.

26th.—Quite well. Discharged cured.

Case III.—David Brewer, æt. seventeen months.

June 19th.—His mother applied for some medicine for a cough which he laboured under. On examining the abdomen, a very large firm tumor was discovered, occupying the left hypochondriac region, extending to the umbilicus and lower part of the iliac region. She has observed his abdomen become gradually enlarged since he was six months old; pulse 130; skin hot; appetite pretty good; bowels confined; last stools dark coloured.

September 11th.—Calomel in small doses, and the mercurial liniment, were continued up to this period, with little alteration in the tumor.

℞. Infusi Quassiæ ℥iv.; Tinct. Iodinæ gtt. xxxij. M. dentur cochla. duo min. nocte maneque.

18th.—Tumor diminished, and softer; general health improved.

Repet^r mistura.

25th.—Tumor diminishes daily: it is now but little apparent, after a careful examination of the soft parts. General health good.

Cont^r medicamenta.

October 2d.—Tumor has entirely disappeared; general health good. Discharged cured.

In the cases above related (in the Medical Repository,) enormous enlargements of the liver and spleen were speedily removed by iodine, given internally, in the form of tincture, after mercury had been given without success. The general health of all the children improved under its use; and, in the case No. 1, incontinence of urine, of long standing, was cured by it.

Iodine appears to possess considerable tonic powers; being a still more powerful stimulant than mercury. It acts as a stimulus to every sensible and moving fibre of the body. In the chronic inflammation particularly, which affects the glandular organs, it is a powerful remedy in counteracting it, and in removing that state of morbid structure which is often its consequence.

Like other powerful remedies, it may no doubt be abused: this, however, is no argument against its properties. Its administration requires to be conducted with much care, being occasionally intermitted, and not too long persisted in; and immediately relinquished if it occasion nausea or purging, vertigo or headache, which are the only bad effects Dr. Milligan observed from its use. When administered to females, its effects should be well watched, as cases are recorded where the mammæ have been entirely absorbed during its long-continued use. But where the removal of a dangerous disease becomes paramount to every other consideration, as in cases of an enlarged thyroid gland pressing on the trachea, and threatening suffocation; and other enlargements, particularly of the scrofulous kind, which other remedies have failed to relieve, the symmetry of the female must become a secondary consideration, and leave little doubt of the propriety of having recourse to iodine. Its good effects, in these cases of very young children just related, go to establish the safety and propriety of the remedy, and afford striking proofs of the advantage to be derived from it.

